# 329. Bedside Inoculation of Blood Culture Bottles Does Not Improve Ascites Culture Positivity Rate in Spontaneous Bacterial Peritonitis

**DOI:** 10.1093/ofid/ofac492.407

**Published:** 2022-12-15

**Authors:** Tyler Brehm, Mayar Al Mohajer, Todd M Lasco

**Affiliations:** Baylor College of Medicine, Houston, Texas; Baylor College of Medicine, Houston, Texas; Baylor St. Luke's Medical Center, Houston, Texas

## Abstract

**Background:**

Prior research demonstrates increased positivity rate for ascites cultures in spontaneous bacterial peritonitis (SBP) when directly inoculated into blood culture bottles at bedside, versus the “conventional method” - transportation of ascitic fluid in a sealed syringe to the laboratory, centrifuging, and plating on Chocolate, Blood, and MacConkey’s agars and in a Schaedler broth. We hypothesized that collection of ascites via direct inoculation into blood culture bottles would improve ascites culture positivity rate in SBP when compared to prior methods.

**Methods:**

In November of 2021, our institution implemented a policy in which all ascites cultures were directly inoculated into blood culture bottles at bedside. We retrospectively reviewed all ascites cultures collected from May of 2021 through April of 2022, including all cultures with an absolute neutrophil count of greater than 250 cells/mm3. We excluded all samples from patients with secondary bacterial peritonitis and all samples collected in November. If a patient had multiple samples that met inclusion criteria, only the first was included for analysis. Our primary outcome was positivity rate of cultures pre- and post-implementation of this new policy.

**Results:**

1375 ascites cultures were collected. Of these, 52 met our inclusion criteria. These patients were on average 58 years old (range 33-74), 50% female, with a median MELD of 25.5 (range 8-40). The conventional method was positive in 7/37 (18.9%) cultures, and direct inoculation of blood cultures in 1/15 (6.67%), p = .27.

**Conclusion:**

As opposed to the previous literature, we detected no difference in positivity rate between the conventional method of ascites culture compared to direct inoculation of blood cultures at the bedside. This difference from prior literature may be due to differences in time to inoculation, our smaller sample size, or overfilling of the bottles during inoculation.

**Disclosures:**

**All Authors**: No reported disclosures.

